# The Tishomingo Medical Association of Mississippi

**Published:** 1874-03

**Authors:** 


THE TISHOMINGO MEDICAL ASSOCIATION OF MISSISSIPPI.—We are pleased to learn of the prosperity of this Association of medical gentlemen. Dr. J. M. Taylor, of Corinth, Mississippi, delivered, March 4, 1874, an elegant and sensible address before the body. Dr. T. is the President of the Mississippi Medical Association, and an accomplisbed gentleman. We have reason to believe the Tishomingo Medical Association will do great things in the interest of our profession, and we wish it and its members the utmost success,

